# Psychometric Properties of the Cognitive Emotion Regulation Questionnaire (CERQ) in Patients with Fibromyalgia Syndrome

**DOI:** 10.3389/fpsyg.2017.02075

**Published:** 2017-12-13

**Authors:** Albert Feliu-Soler, Elvira Reche-Camba, Xavier Borràs, Adrián Pérez-Aranda, Laura Andrés-Rodríguez, María T. Peñarrubia-María, Mayte Navarro-Gil, Javier García-Campayo, Juan A. Bellón, Juan V. Luciano

**Affiliations:** ^1^Institut de Recerca Sant Joan de Déu, Barcelona, Spain; ^2^Teaching, Research & Innovation Unit, Parc Sanitari Sant Joan de Déu, St. Boi de Llobregat, Spain; ^3^Primary Care Prevention and Health Promotion Research Network, RedIAPP, Madrid, Spain; ^4^Facultat de Psicologia, Universitat Autònoma de Barcelona, Barcelona, Spain; ^5^Primary Health Centre Bartomeu Fabrés Anglada, DAP Costa de Ponent, Institut Català de la Salut- IDIAP Jordi Gol, Gavà, Spain; ^6^Centre for Biomedical Research in Epidemiology and Public Health CIBERESP, Madrid, Spain; ^7^Faculty of Psychology, University of Zaragoza, Zaragoza, Spain; ^8^Instituto de Investigaciones Sanitarias, Psychiatry Service, Hospital Universitario Miguel Servet, Zaragoza, Spain; ^9^Primary Care Center El Palo, Málaga, Spain; ^10^Department of Preventive Medicine, Public Health and Psychiatry, University of Málaga, Málaga, Spain

**Keywords:** cognitive emotion regulation questionnaire (cerq), fibromyalgia, pain, depression, confirmatory factor analysis

## Abstract

Given that Fibromyalgia Syndrome (FMS) is associated with problems in emotion regulation, the importance of assessing this construct is widely acknowledged by clinical psychologists and pain specialists. Although the Cognitive Emotion Regulation Questionnaire (CERQ) is a self-report measure used worldwide, there are no data on its psychometric properties in patients with FMS. This study analyzed the dimensionality, reliability, and validity of the CERQ in a sample of 231 patients with FMS. Given that “fibrofog” is one of the most disabling FMS symptoms, in the present study, items in the CERQ were grouped by dimension. This change in item presentation was conceived as an efficient way of facilitating responses as a result of a clear understanding of what the items related to each dimension are attempting to measure. The following battery of measures was administered: the CERQ, the Revised Fibromyalgia Impact Questionnaire, the Pain Catastrophizing Scale, the Center for Epidemiologic Studies Depression Scale, and the State-Trait Anxiety Inventory. Four models of the CERQ structure were examined and confirmatory factor analyses supported the original factor model, consisting of nine factors—Self-blame, Acceptance, Rumination, Positive refocusing, Refocus on planning, Positive reappraisal, Putting into perspective, Catastrophizing, and Other-blame. There was minimal overlap between CERQ subscales and their internal consistency was adequate. Correlational and regression analyses supported the construct validity of the CERQ. Our findings indicate that the CERQ (items-grouped version) is a sound instrument for assessing cognitive emotion regulation in patients with FMS.

## Introduction

Chronic pain conditions, such as fibromyalgia syndrome (FMS), are amongst the most common health problems managed by general practitioners, rheumatologists, and clinical psychologists (Häuser et al., 2015). FMS is characterized by multifocal pain, fatigue, non-restorative sleep, cognitive complaints (also known as *fibrofog*: lack of attention-concentration, decrease in memory, and loss of vocabulary, which are exacerbated in stressful situations), high levels of distress, and is associated with greater affect intensity, which in turn correlates with more pain and fatigue in those patients with deficient emotion processing skills (van Middendorp et al., 2008; Geenen et al., 2012). Emotion regulation refers to “*the extrinsic and intrinsic processes responsible for monitoring, evaluating and modifying emotional reactions, especially their intensive and temporal features, to accomplish one's goals*” (Thompson, 1994, p. 27). According to van Middendorp et al. (2008), the strategies to regulate unpleasant emotions such as sadness or anger play an important role in the maintenance or exacerbation of FMS symptoms. Moreover, impaired emotion regulation is a transdiagnostic risk factor that has been implicated in many disorders, including those related to mood, anxiety, substance use, personality, and eating (Naragon-Gainey et al., 2017). Emotion regulation strategies have been incorporated into some models of psychopathology and various therapeutic approaches (Aldao et al., 2010). For instance, Catastrophizing is a critically important risk factor for adverse pain-related outcomes and is directly associated with amplification of pain processing in the brain, whereas Reappraisal has a beneficial impact on an individual's emotional state. In the long-term, it reduces chronic arousal of the hypothalamic-pituitary-adrenal axis (Edwards et al., 2009; Malfliet et al., 2017).

Hence, the availability of conceptually and psychometrically sound measures of *emotion reactivity* (how readily one experiences an emotion, how intensely, and for how long) and *emotion regulation* is an important component in the comprehensive assessment of patients in clinical research and practice (Zelkowitz and Cole, 2016). Focusing on the self-regulatory, conscious, cognitive components of emotion regulation, Garnefski et al. (2001) developed the Cognitive Emotion Regulation Questionnaire (CERQ). The authors revised existing measures to take out or reformulate items capturing cognitive dimensions, to transform non-cognitive coping strategies into cognitive dimensions, and to add new strategies taking into account rational grounds. The CERQ is a 36-item self-report measure that captures stable-dispositional cognitive emotion regulation strategies when people experience stressful or threatening life experiences. Specifically, the following strategies are measured: Self-blame, Blaming others, Acceptance, Refocusing on planning, Positive refocusing, Rumination, Positive reappraisal, Putting into perspective, and Catastrophizing. *Self-blame and Blaming others* are the cognitive strategies which refer to causal attribution of the negative event to oneself or the others; *Rumination* consists in overthinking about the consequences of the negative event; *Catastrophizing* is described as anticipating thoughts about exaggerated consequences of the negative event; on the other hand, *Putting into perspective* refers to relativizing the unpleasant event by comparing it to others or considering its impact over time; *Positive refocusing* consists of trying to keep the attention on pleasant thoughts after the occurrence of a negative situation; *Positive reappraisal*, is the strategy by which the individual tries to find the silver lining in the negative event; *Acceptance* refers to the cognitive process by which the individual stops trying to change the negative situation or the emotions that it caused and just experiences them; finally, *Planning* is described as the strategy by which the attention is focused on what the individual can do to solve the unpleasant situation or make it easier to deal with. A detailed explanation of the cognitive strategies is provided in the pioneer study by Garnefski et al. (2001).

When adults from a clinical sample with clinically relevant depression and anxiety, and subjects from a matched non-clinical sample both completed the CERQ, Garnefski et al. (2002) found Cronbach's α values that ranged from 0.72 (Acceptance) to 0.85 (Self-blame). For cognitive research to remain linked to clinical practice, it is crucial for instruments to perform well in both clinical and non-clinical samples. Garnefski et al. (2002) found significant differences between the clinical and the non-clinical groups in Catastrophizing, Self-blame, Rumination, Other-blame, Positive reappraisal, and Acceptance. Of these strategies, only Positive reappraisal appeared to be reported significantly more often by the non-clinical group than by the clinical group. Garnefski and Kraaij (2006) compared early adolescent, late adolescent, adult, elderly and psychiatric samples on their reported use of cognitive emotion regulation strategies. As expected, data analyses revealed significantly higher scores for Self-blame, Rumination, Catastrophizing and Other-blame in the adult psychiatric sample, supporting the construct validity of the CERQ. In another study, Garnefski and Kraaij (2007) reported adequate goodness-of fit values for the nine-factor model (CFI = 0.92 and 0.97 in two different time points), which confirmed the robustness of the CERQ factor structure.

The CERQ has been translated and validated into many languages and cultures, such as French (Jermann et al., 2006), Chinese (Zhu et al., 2008), Turkish (Tuna and Bozo, 2012), Persian (Abdi et al., 2012), Spanish (Domínguez-Sánchez et al., 2013; Medrano et al., 2013; Domínguez-Lara and Medrano, 2016) and Arabic (Megreya et al., 2016), showing adequate reliability and validity. A recent cross-cultural study (Potthoff et al., 2016) compared CERQ scores across six European countries (Netherlands, Hungary, Spain, Italy, Portugal, and Germany) using general population samples, all comparable in terms of age and educational backgrounds. Although some between-country differences were observed in subscale scores, there was a consistent link between cognitive emotion regulation strategies and psychopathology. More recently, Ireland et al. (2017) examined the dimensionality, and construct validity of the CERQ, both short (18 items) and long (36 items) form, in 795 community residents evaluated online. Although model fit was better for the 18-item CERQ, the correlational analyses with difficulties in emotion regulation and positive/negative affect values indicated a statistically significant small to medium drop in variance explained by the CERQ-short when compared with the full CERQ, which suggests better convergent validity for the full version of the instrument. To sum up, the CERQ seems to be an optimal candidate for the assessment of emotion regulation in clinical and non-clinical samples.

To date, none of the published studies on the CERQ has examined the psychometric properties of the instrument in patients with FMS. Verification of the original nine-factor model, as well as of adequate reliability and validity in these patients, is lacking. Taking this as its foundation, the present study examines the internal consistency and convergent-discriminant validity of the Spanish CERQ and evaluates its dimensionality using confirmatory factor analysis (CFA) in a pooled sample of patients with FMS. In line with previous studies, a nine-factor solution in addition to unidimensional and hierarchical factor solutions were tested. We expected that the original nine-factor model would provide the best fit. Second, the internal consistency (Cronbach's α) of the best fitting factor structure of the CERQ was determined. Third, construct validity (convergent validity) of the best fitting factor structure of the CERQ was assessed by investigating the relationships with self-report measures of psychological symptoms (anxiety and depression) and pain-related constructs such as pain catastrophizing and functional status in FMS. Given that depression is a disorder characterized by impaired emotion regulation (Joormann and Stanton, 2016), we compared the CERQ scores of subgroups of FMS patients with distinct levels of depressive symptoms to establish the discriminant validity of the CERQ.

## Materials and methods

In the present study, we utilized the dataset from the Fibromyalgia Subtypes study (Luciano et al., 2016) and early-stage data from the EUDAIMON study (Feliu-Soler et al., 2016). Study data are available from the corresponding author. Written informed consent was obtained from patients of both studies. Table 1 displays participant characteristics for the two samples.

**Table 1:** Participant Characteristics for the Two Samples and the Entire Sample.

**Socio-demographic variables**	**Sample 1 (*n* = 160)**	**Sample 2 (*n* = 71)**	**Total sample (*n* = 231)**
Gender (*n* females, %)	156 (97.5)	71 (100)	227 (98.3)
Age, M (SD)	57.28 (8.8)	52.63 (7.2)	55.89 (8.6)
**Marital status**, ***n*** **(%)**			
*Single*	5 (3.1)	3 (4.2)	8 (3.5)
*Married/Living with a partner*	118 (73.8)	57 (80.3)	175 (76.1)
*Separated/divorced*	20 (12.5)	9 (12.7)	29 (12.6)
*Widowed*	17 (10.6)	1 (1.4)	18 (7.8)
**Living arrangements**, ***n*** **(%)**			
*Living alone*	18 (11.3)	1 (1.4)	19 (8.2)
*Living with someone (spouse/partner/relatives)*	142 (88.8)	69 (97.2)	211 (91.3)
**Educational level**, ***n*** **(%)**			
*No formal education*	33 (20.6)	1 (1.4)	33 (14.3)
*Did not graduate from primary school*	30 (18.8)	2 (2.8)	32 (13.9)
*Primary school*	56 (35)	36 (50.7)	92 (39.8)
*Secondary school*	35 (21.9)	30 (42.3)	65 (28.1)
*University*	6 (3.8)	2 (2.8)	8 (3.5)
**Work status**, ***n*** **(%)**			
*Homemaker*	40 (25)	9 (12.9)	49 (21.3)
*Paid employment*	25 (15.6)	23 (32.9)	48 (20.8)
*Paid employment but on sick leave*	7 (4.4)	6 (8.6)	13 (5.7)
*Unemployed with allowance*	25 (15.6)	6 (8.6)	31 (13.4)
*Unemployed without allowance*	14 (8.8)	11 (15.7)	25 (10.8)
*Retired/pensioner*	25 (15.6)	7 (10)	32 (13.9)
*Temporarily disabled*	–	1 (1.4)	1 (0.4)
*Others (e.g., student)*	24 (15)	7 (10)	31 (13.8)
**Clinical variables, M (SD)**			
FIQ-R (0-100)	68.90 (18.87)	59.41 (21.23)	65.99 (20.07)
*Function* (0-30)	20.54 (6.67)	18.20 (6.50)	19.81 (6.70)
*Overall impact* (0-20)	12.79 (7.33)	9.29 (7.31)	11.71 (7.49)
*Severity of symptoms* (0-50)	35.58 (8.07)	31.91 (9.83)	34.45 (8.80)
PCS (0-52)	31.47 (14.06)	21.63 (13.25)	28.48 (14.52)
*Rumination* (0–16)	10.36 (4.82)	7.70 (4.93)	9.55 (4.98)
*Magnification* (0-12)	6.49 (3.43)	4.23 (2.92)	5.80 (3.44)
*Helplessness* (0-24)	14.63 (7.08)	9.70 (6.70)	13.13 (7.32)
CES-D (0-60)	34.34 (11.79)	–	–
STAI-T (0-60)	37.50 (10.56)	–	–

*CES-D, Center for Epidemiologic Studies Depression Scale; FIQ-R, Fibromyalgia Impact Questionnaire Revised; PCS, Pain Catastrophizing Scale; STAI-T, Trait Anxiety Inventory*.

Sample 1 (Fibromyalgia Subtypes study) consisted of a convenience sample of 160 adult patients with FMS recruited from 14 physician practices within the Barcelona metropolitan area (Spain). The family physicians at these centers referred suspected FMS cases to Viladecans Hospital or Sant Joan de Déu Hospital (the two reference hospitals in the area). Rheumatologists from these hospitals confirmed or ruled out the diagnosis of FMS following American College of Rheumatology (ACR) 1990 criteria (Wolfe et al., 1990), and added the patients to a database if they received a FMS diagnosis. Adult patients (≥18 years-old) in these databases were candidates for inclusion in the study. A detailed description of the study protocol and inclusion/exclusion criteria can be found elsewhere (Luciano et al., 2016). The study protocol was approved by the Ethics Committee at the Sant Joan de Déu Foundation (CEIC PIC-33-11; Esplugues de Llobregat, Spain) and by the Jordi Gol i Gurina Foundation research ethics committee (P12/94; Barcelona, Spain).

Sample 2 consisted of 71 patients with FMS recruited for the EUDAIMON study. This ongoing study is a 12-month, randomized controlled trial, the main aim of which is to assess the effectiveness and cost-utility of a mindfulness-based intervention for FMS patients compared with a psycho-educational intervention (FibroQoL) and treatment as usual. For the present work, we used only the EUDAIMON baseline dataset. Patients were selected following a multi-stage recruitment process. All recruited patients are adults diagnosed with FMS according to the ACR 1990 by rheumatologists from the Sant Joan de Déu Hospital. A detailed description of the study protocol and inclusion/exclusion criteria can be found elsewhere (Feliu-Soler et al., 2016). The RCT is being performed in accordance with ethical standards laid down in the 1964 Declaration of Helsinki and its subsequent updates. The Ethics Committee at the Sant Joan de Déu Foundation evaluated and approved the study protocol in May 2015 (PIC-102-15).

### Procedure

In both studies (Feliu-Soler et al., 2016; Luciano et al., 2016), a randomized list of potential participants was delivered to a research assistant (health psychologist) who screened patients through a phone interview until the targeted sample size was achieved. The research assistant then made an appointment for those patients that agreed to participate in the study. In the Fibromyalgia Subtypes study (Luciano et al., 2016), the research assistant performed all the face-to face interviews in the general practices or in the reference hospitals once written consent had been obtained, whereas in the EUDAIMON study (Feliu-Soler et al., 2016), the CERQ was completed at home and collected by the research assistant (blind to group allocation) on the participants' following visit to the hospital (1–2 weeks later).

### Study measures

Participants from both studies completed the following paper-and-pencil measures:

The Socio-Demographic questionnaire collected information on the following variables: gender, date of birth, marital status, living arrangements, educational level, employment status, type of contract (question for employees), and years since FMS diagnosis.

The *Cognitive Emotion Regulation Questionnaire* (CERQ; Garnefski et al., 2001) is a 36-item self-report measure designed to assess individual differences in cognitive regulation of emotions in response to stressful, threatening or traumatic life events. The instrument assesses nine 4-item dimensions: Self-blame, Blaming others, Acceptance, Refocusing on planning, Positive refocusing, Rumination, Positive reappraisal, Putting into perspective, and Catastrophizing. Responses are given on a 5-point Likert scale ranging from 1 “(almost) never” to “(almost) always.” Therefore, subscale scores can range from 4 to 20 with higher subscale scores indicating greater frequency of use of the specific cognitive strategy. Regarding the Spanish version, it was tested in a large non-clinical sample (*n* = 615 students) by Domínguez-Sánchez et al. (2013), who obtained a hierarchical structure composed of nine dimensions distributed into two second-order factors (adaptive strategies and less adaptive strategies). The internal consistency, test-retest reliability and criterion validity were adequate or acceptable. A characteristic of the CERQ, in common with most multidimensional instruments, is that items are not grouped by dimension, but are dispersed throughout the instrument. Specifically, the questionnaire developers chose a rotating selection strategy, so that every ninth item is presupposed to belong to the same dimension. For instance, items 1, 10, 19, and 28 are considered to belong to Self-blame. Given that *fibrofog* is one of the most prominent FMS symptoms, in this study, items in the CERQ were grouped (but not labeled) by dimension. This change in item presentation was conceived as an efficient way of facilitating responses as a result of a clear understanding of what the items related to each dimension are attempting to measure (Schell and Oswald, 2013). Thus, we expected to have an instrument perfectly aligned with our target sample that could provide more trustworthy information about emotion regulation with the confidence that there is available empirical evidence that item order, within honest conditions (when faking is not presupposed), does not alter the underlying measurement properties of psychological instruments (Schell and Oswald, 2013).

The *Revised Fibromyalgia Impact Questionnaire* (FIQR; Bennett et al., 2009; Luciano et al., 2013) is the recommended instrument for measuring functional status in FMS patients. It includes 21 items that are all answered on an 11-point numeric rating scale of 0-to-10, with 10 reflecting greater impairment. The time frame is the previous 7 days, with the items distributed across three associated domains: “function” (9 items); “overall impact” (2 items); and “severity of symptoms” (10 items). The scoring system is as follows: the physical function domain (0-to-90) is divided by 3, the overall impact domain (0-to-20) is not transformed, and the severity of symptoms domain (0-to-100) is divided by 2. FIQR reliability in our pooled sample was good (Cronbach's α = 0.89).

The *Pain Catastrophizing Scale* (PCS; Sullivan et al., 1995; García-Campayo et al., 2008) is a 13-item instrument that consists of 3 dimensions: Rumination (tendency to focus excessively on pain sensations), Magnification (tendency to magnify the threat value of pain sensations), and Helplessness (tendency to perceive oneself as unable to control the intensity of pain). The PCS total score and subscale scores are computed as the algebraic sum of ratings for each item. PCS items are rated in relation to the frequency of occurrence on 5-point scales (0 = never, 4 = almost always), and total scores can vary from 0 to 52. Higher scores indicate greater pain catastrophizing. Internal consistency was excellent in the pooled sample (Cronbach's α = 0.94).

In addition, the participants from the Fibromyalgia Subtypes study completed the following paper-and-pencil measures:

The *Center for Epidemiologic Studies Depression Scale* (CES-D; Radloff, 1977; Vázquez et al., 2007) is a 20-item scale frequently used to assess depressive symptom severity. The time frame is the previous week. Item responses range from 0 to 3 [0 = rarely or none of the time (<1 day in the past week), 1 = some or a little of the time (1–2 days), 2 = occasionally or a moderate amount of the time (3–4 days), and 3 = most or all of the time (5–7 days)]. Therefore, total scores can vary from 0 to 60, with higher scores reflecting increased depression severity. The CES-D has been widely used to detect mood disturbances in many populations, including FMS patients, demonstrating adequate psychometric properties (Smarr and Keefer, 2011). A recent meta-analysis (Vilagut et al., 2016) focused on CES-D screening accuracy for depression observed that a cut-off score ≥20 yielded the best trade-off between sensitivity (0.83) and specificity (0.78). The CES-D had high internal consistency (Cronbach's α = 0.86).

The *Spanish State-Trait Anxiety Inventory* (STAI—form X; Spielberger et al., 1986) is a 40-item, self-report measure of general anxiety. The first 20 items (STAI-S) measure state anxiety, or how the subject feels right now. The second 20 items (STAI-T) assess trait anxiety, or how the subject generally feels. We only used the STAI-T. Individuals have to rate each item using a Likert-type scale from 0 (not at all) to 3 (very much so). Total scores on the STAI-T vary from 0 to 60, with higher scores indicating more trait anxiety. Cronbach's α for the STAI-T was 0.84.

### Statistical analyses

SPSS v22.0 and MPlus v7.4 were used to compute the data analyses.

First, we conducted a CFA to test the fit of the following factor structures: the one-factor model with all CERQ items loading on one latent factor, the original nine-factor model by Garnefski et al. (2001) with Self-blame, Other-blame, Catastrophizing, Rumination, Acceptance, Positive refocusing, Refocus on planning, Positive reappraisal, and Putting into perspective. Finally, we tested the higher order factor model reported by Domínguez-Sánchez et al. (2013) with the nine dimensions grouped into two general latent dimensions of *adaptive strategies* (Acceptance, Positive refocusing, Refocus on planning, Positive reappraisal, Putting into perspective) and *less adaptive strategies* (Self-blame, Rumination, Catastrophizing, and Other-blame). In ordinal items with a non-normal distribution, such as those in the CERQ, it may be expected that the covariance matrix will underestimate the true extent of relationships among items. Therefore, we proceeded to estimate the models from the polychoric correlation matrix. Mean and Variance corrected Weighted Least Squares (WLSMV) was applied to test the fit of the three factor models. The following indices were examined to evaluate model fit: χ^2^ (a non-significant estimate reflects good fit), the Tucker-Lewis Index (TLI ≥0.90), the comparative fit index (CFI ≥ 0.90), and the root means square error of approximation (RMSEA ≤ 0.08).

Second, we calculated the internal consistency for each CERQ domain by computing Cronbach's α in the pooled sample. A common rule of thumb criterion is a Cronbach's α of 0.6 for exploratory research and of 0.7 for confirmatory research (Hair et al., 1998). In addition, we assessed homogeneity of the CERQ subscales by inspecting the corrected item total correlation (correlation of the designated item with the total score for all other subscale items). A cut-off score of 0.3 is recommended for the corrected item-total correlations (Nunnally and Bernstein, 1994).

Third, we examined the correlations among the CERQ subscales as well as their construct validity by computing Pearson product moment correlations between each of the CERQ subscales with the measures of functional status (FIQR), pain catastrophizing (PCS), depressive symptoms (CES-D), and trait anxiety (STAI-T). We took Cohen (1988) into account to evaluate the substantive significance of correlations (large correlations are those >0.5, medium correlations are from 0.3 to 0.49, and small correlations are from 0.1 to 0.29).

Finally, the known-groups' validity approach is founded on the hypothesis that specific subgroups of patients might be expected to score differently from others. In this study, a set of Student's *t*-tests for independent samples was computed to assess the validity of the CERQ subscales to discriminate between the FMS patients with clinically relevant depressive symptoms and those without (according to the CES-D cut-off value ≥20; Vilagut et al., 2016). We calculated between-groups effect sizes using Cohen's d with a 95% confidence interval. The rule of thumb for Cohen's d is that 0.2 is small, 0.5 is medium, and 0.8 is large. Additionally, bearing in mind that the separate cognitive emotion regulation strategies have overlapping processes and due to the likely significant subscale intercorrelations, multivariate analyses accounting for the intercorrelations are needed to identify unique relationships between cognitive emotion regulation strategies and clinical subgroup membership (FMS with vs. without depression). Therefore, we computed a logistic regression analysis to examine the unique “influence” of each strategy on subgroup membership, while controlling for the influence of the other strategies (Garnefski et al., 2002). In this analysis, the binary dependent variable was subgroup membership (FMS with vs. without depression), whereas the independent variable set consisted of the nine cognitive emotion regulation strategies.

## Results

### Testing competing confirmatory factor analytic CERQ models

In the CFA involving the one-factor model, we found that it provided a very poor fit to the sample data: $\chi_{\left(594,\;\text{N}=229\right)}^{2}$ = 5,564.958, *p* < 0.001, CFI = 0.527, TLI = 0.498, and RMSEA = 0.191 (90% CI, 0.187–0.196). Consistent with Garnefski et al. (2001), a nine-factor model adequately fit the data, $\chi_{\left(558,\;\text{N}=229\right)}^{2}$ = 1,302.203, *p* < 0.001, CFI = 0.929, TLI = 0.920, and RMSEA = 0.076 (90% CI, 0.071–0.082). Standardized factor loadings for the nine-factor model were all statistically significant and ranged from 0.542 (item 29) to 0.957 (item 34). See Table 2 for standardized factor loading estimates. For the sake of comparability, Table 2 also shows factor loadings reported by Garnefski and Kraaij (2007) in a sample of 611 Dutch adults from the general population and by Domínguez-Sánchez et al. (2013) in 615 Spanish students.

**Table 2:** Item Content, Mean (M), Standard Deviation (SD), and Factor Loadings (λ, 9-factor solution) of the CERQ Items.

**Scale names (Cronbach α values) and items**	**Sample 1 M (SD)**	**Sample 2 M (SD)**	**Total sample M (SD)**	**λ**	**Domínguez-Sánchez et al. (2013) λ *n* = 615**	**Garnefski and Kraaij (2007) λ T1 / T2 *n* = 611**
***Self-blame* (α = 0.86)**						
1. I feel that I am the one to blame for it (1)	2.33 (1.27)	2.17 (1.19)	2.33 (1.26)	0.89	0.79	0.70/0.70
2. I feel that I am the one who is responsible for what has happened (10)	2.23 (1.21)	2.14 (1.20)	2.23 (1.20)	0.92	0.68	0.71/0.70
3. I think about the mistakes I have made in this matter (19)	2.98 (1.34)	3.06 (1.28)	2.98 (1.33)	0.73	−0.11	0.55/0.57
4. I think that basically the cause must lie within myself (28)	2.26 (1.24)	2.27 (1.14)	2.26 (1.24)	0.82	0.69	0.80/0.77
***Acceptance* (α = 0.77)**						
5. I think that I have to accept that this has happened (2)	3.36 (1.35)	3.21 (1.29)	3.36 (1.35)	0.93	0.72	0.73/0.77
6. I think that I have to accept the situation (11)	3.47 (1.35)	3.46 (1.27)	3.47 (1.35)	0.96	0.87	0.70/0.71
7. I think that I cannot change anything about it (20)	3.13 (1.43)	2.91 (1.34)	3.13 (1.43)	0.87	−0.10	0.66/0.65
8. I think that I must learn to live with it (29)	3.80 (1.30)	3.51 (1.33)	3.80 (1.29)	0.91	0.61	0.69/0.61
***Rumination* (α = 0.84)**						
9. I often think about how I feel about what I have experienced (3)	3.23 (1.35)	2.97 (1.32)	3.23 (1.34)	0.80	0.71	0.75/0.66
10. I am preoccupied with what I think and feel about what I have experienced (12)	3.19 (1.41)	3.21 (1.35)	3.19 (1.40)	0.82	0.74	0.77/0.74
11. I want to understand why I feel the way I do about what I have experienced (21)	3.23 (1.40)	3.24 (1.43)	3.23 (1.39)	0.70	0.52	0.66/0.69
12. I dwell upon the feelings the situation has evoked in me (30)	3.36 (1.40)	3.25 (1.42)	3.36 (1.40)	0.88	0.75	0.68/0.77
***Positive refocusing* (α = 0.93)**						
13. I think of nicer things than what I have experienced (4)	2.61 (1.40)	2.83 (1.23)	2.61 (1.40)	0.86	0.83	0.76/0.79
14. I think of pleasant things that have nothing to do with it (13)	2.60 (1.48)	2.94 (1.42)	2.60 (1.47)	0.95	0.86	0.85/0.87
15. I think of something nice instead of what has happened (22)	2.27 (1.28)	2.46 (1.23)	2.27 (1.27)	0.93	0.85	0.83/0.80
16. I think about pleasant experiences (31)	2.40 (1.34)	2.82 (1.36)	2.40 (1.33)	0.95	0.91	0.67/0.74
***Refocus on planning* (α = 0.83)**						
17. I think of what I can do best (5)	3.37 (1.26)	3.46 (1.24)	3.37 (1.25)	0.76	0.72	0.69/0.81
18. I think about how I can best cope with the situation (14)	3.30 (1.24)	3.45 (1.08)	3.30 (1.23)	0.84	0.84	0.75/0.80
19. I think about how to change the situation (23)	3.23 (1.31)	3.15 (1.28)	3.23 (1.30)	0.75	0.71	0.74/0.71
20. I think about a plan of what I can do best (32)	3.12 (1.37)	3.08 (1.28)	3.12 (1.37)	0.82	0.81	0.78/0.77
***Positive reappraisal* (α = 0.80)**						
21. I think I can learn something from the situation (6)	3.10 (1.41)	2.89 (1.30)	3.10 (1.40)	0.74	0.83	0.67/0.72
22. I think that I can become a stronger person as a result of what has happened (15)	2.74 (1.44)	2.65 (1.36)	2.74 (1.44)	0.71	0.81	0.59/0.59
23. I think that the situation also has its positive sides (24)	2.65 (1.44)	2.46 (1.41)	2.65 (1.44)	0.78	0.79	0.64/0.52
24. I look for the positive sides to the matter (33)	2.85 (1.47)	2.63 (1.40)	2.85 (1.46)	0.86	0.94	0.73/0.70
***Putting into perspective* (α = 0.79)**						
25. I think that it all could have been much worse (7)	3.10 (1.36)	2.89 (1.35)	3.10 (1.35)	0.59	0.68	0.62/0.60
26. I think that other people go through much worse experiences (16)	3.64 (1.37)	3.14 (1.42)	3.64 (1.36)	0.82	0.80	0.77/0.79
27. I think that it hasn't been too bad compared to other things (25)	2.96 (1.31)	2.91 (1.26)	2.96 (1.31)	0.82	0.87	0.68/0.79
28. I tell myself that there are worse things in life (34)	3.48 (1.37)	3.24 (1.34)	3.48 (1.37)	0.80	0.81	0.70/0.80
***Catastrophizing* (α = 0.82)**						
29. I often think that what I have experienced is much worse than what others have experienced (8)	2.16 (1.27)	2.03 (1.08)	2.16 (1.26)	0.55	0.46	0.75/0.34
30. I keep thinking about how terrible it is what I have experienced (17)	2.39 (1.30)	2.30 (1.19)	2.39 (1.30)	0.85	0.87	0.64/0.75
31. I often think that what I have experienced is the worst that can happen to a person (26)	2.07 (1.26)	1.86 (1.14)	2.07 (1.26)	0.79	0.63	0.70/0.80
32. I continually think how horrible the situation has been (35)	2.42 (1.32)	2.13 (1.13)	2.42 (1.31)	0.91	0.81	0.59/0.78
***Other-blame* (α = 0.92)**						
33. I feel that others are to blame for it (9)	1.77 (1.24)	1.51 (0.95)	1.77 (1.24)	0.93	0.81	0.75/0.71
34. I feel that others are responsible for what has happened (18)	1.80 (1.20)	1.52 (0.89)	1.80 (1.19)	0.96	0.79	0.82/0.79
35. I think about the mistakes others have made in this matter (27)	2.22 (1.31)	1.81 (1.08)	2.22 (1.30)	0.87	0.50	0.72/0.72
36. I feel that basically the cause lies with others (36)	1.82 (1.27)	1.54 (1.00)	1.82 (1.27)	0.91	0.87	0.83/0.81

*Original item numbering is presented between brackets. T1 = Time 1; T2 = Time 2*.

**Figure 1:**
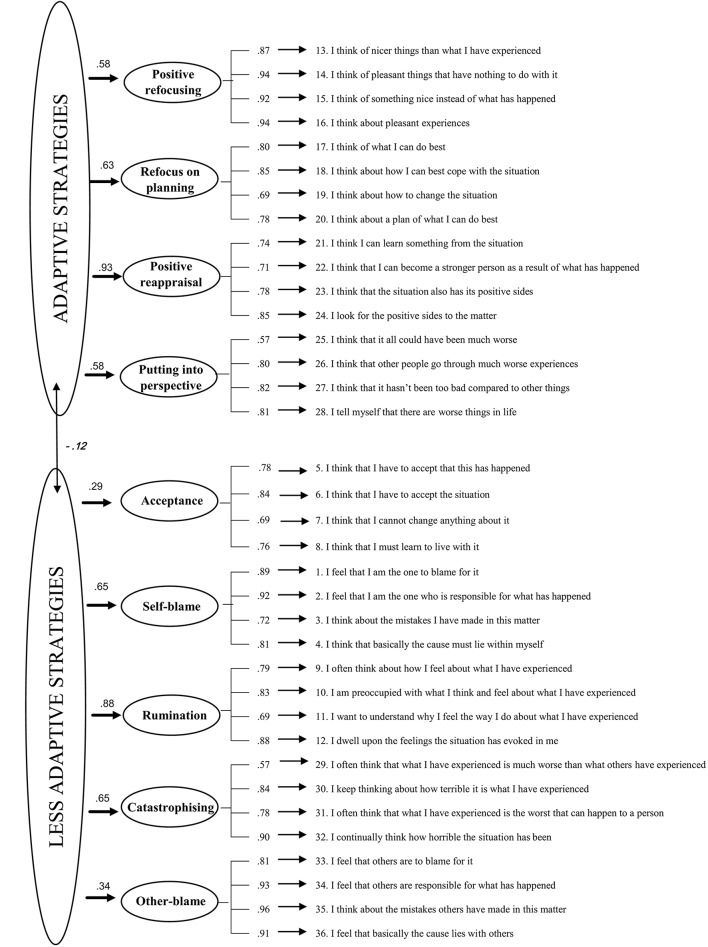
Standardized factor loadings in the hierarchical model with Acceptance as part of the “less adaptive” strategies second-order factor. Non-significant values are given in Italics.

The hierarchical factor model revealed that the inclusion of two second-order factors (adaptive and less adaptive strategies) produced a worse fit to the data compared to the nine-factor model, $\chi_{\left(584,\;\text{N}=229\right)}^{2}$ = 1,519.054, *p* < 0.001, CFI = 0.911, TLI = 0.904, and RMSEA = 0.084 (90% CI, 0.078 −0.089). One of the reasons for the worse fit was the low factor loading (λ = 0.135, *p* = 0.044) of Acceptance with the second-order factor labeled as adaptive strategies. Therefore, we tested a respecification of the second-order factor model that incorporated Acceptance on the latent factor labeled as less adaptive strategies. This hierarchical model showed a slightly better fit across all indices, compared with the previously estimated hierarchical model $\chi_{\left(584,\;\text{N}=229\right)}^{2}$ = 1462.583, *p* < 0.001, CFI = 0.916, TLI = 0.910, and RMSEA = 0.081 (90% CI, 0.076 −0.086). The Acceptance dimension was more strongly related to the less adaptive strategies latent factor (λ = 0.287, *p* < 0.001) than with the adaptive strategies factor. For illustrative purposes, the second hierarchical model is displayed in Figure 1. Therefore, we decided to retain the nine CERQ domains for further analyses (reliability and validity) given that, among the tested models, the first-order nine-factor model showed the best fit to the data and because of parsimony considerations^1^.

### Reliability and homogeneity of the CERQ subscales

As can be seen in Table 2, Cronbach's α reliability scores for the CERQ subscales in FMS patients ranged from 0.77 (Acceptance) to 0.93 (Positive refocusing) and the values of the corrected item-total correlations ranged from 0.44 (item 25) to 0.87 (items 14, 16, and 34). The average corrected item-total correlation was *r* = 0.7 (Self-blame), 0.58 (Acceptance), 0.67 (Focus on thoughts), 0.85 (Positive refocusing), 0.67 (Refocus on planning), 0.61 (Positive reappraisal), 0.6 (Putting into perspective), 0.64 (Catastrophizing), and 0.81 (Other-blame). Squaring that value shows that 49, 34, 45, 72, 45, 37, 36, and 41% of the variance of the average item overlaps with the remaining subscale items, respectively.

### Intercorrelations among the CERQ subscales

As displayed in Table 3, correlations among the CERQ subscales fell between non-significant (n.s) and one large value (0.54 for Self-blame and Rumination). Notably, half of the computed correlations (18/36) were not statistically significant. The majority of the significant relationships were small or medium in magnitude, suggesting that the subscales are relatively independent. Following Cohen's (1988) criteria to evaluate the substantive significance of correlations, the average size of the significant intercorrelations found among the adaptive and less adaptive subscales was medium in both cases (*r* = 0.38 and 0.33, respectively).

**Table 3:** Intercorrelations among the CERQ Subscales.

***CERQ Subscales***	**SB**	**A**	**RUM**	**PR**	**RP**	**POSR**	**PP**	**CAT**	**OB**
Self-Blame (SB)	–	0.25^**^	0.54^**^	−0.29^**^	0.17^**^	n.s	n.s.	0.29^**^	n.s.
Acceptance (A)		–	0.28^**^	n.s.	n.s.	n.s.	n.s.	n.s.	n.s.
Rumination (RUM)			–	−0.21^**^	0.26^**^	n.s.	n.s.	0.42^**^	0.18^**^
Positive refocusing (PR)				–	0.31^**^	0.41^**^	0.24^**^	−0.21^**^	n.s.
Refocus on planning (RP)					–	0.48^**^	0.35^**^	n.s.	n.s
Positive reappraisal (POSR)						–	0.47^**^	n.s.	n.s
Putting into perspective (PP)							–	n.s.	n.s
Catastrophizing (CAT)								–	0.38^**^
Other-Blame (OB)									–

n.s., non-significant;

^*^p < 0.05;

** *p < 0.01*.

### Convergent validity: association of the CERQ subscales with study measures

The results are shown in Table 4. On the one hand, it is interesting to note that Acceptance presented significant, positive, small correlations with the CES-D and STAI-T and the FMS-related measures (FIQR and PCS) as well, which supported the second-order factor model reported above. In a similar vein, the other less adaptive strategies (Self-blame, Rumination, Catastrophizing, and Other-blame) showed a significant pattern of positive correlations with the study measures. On the other hand, two of the adaptive CERQ strategies (Refocus on planning and Putting into perspective) presented null correlations with the study measures. Only Positive refocusing and Positive reappraisal presented the expected significant negative relationships with trait anxiety, depression symptoms, functional impairment and pain catastrophizing. All these correlations were of small magnitude with the exception of those obtained by Positive refocusing with depressive symptoms and trait anxiety, which were medium-to-large.

**Table 4:** Intercorrelations between the CERQ Subscales and Study Measures.

***CERQ Subscales***	**FIQR**	**PCS**	**CES-D**	**STAI-T**
Self-Blame	0.31^**^	0.35^**^	0.42^**^	0.45^**^
Acceptance	0.29^**^	0.21^**^	0.18^*^	0.21^**^
Rumination	0.34^**^	0.32^**^	0.39^**^	0.46^**^
Positive refocusing	−0.14^*^	−0.24^**^	−0.48^**^	−0.55^**^
Refocus on planning	n.s.	n.s.	n.s.	n.s.
Positive reappraisal	−0.14^*^	−0.13^*^	−0.26^**^	−0.32^**^
Putting into perspective	n.s.	n.s.	n.s.	n.s.
Catastrophizing	0.39^**^	0.41^**^	0.47^**^	0.43^**^
Other-Blame	0.14^*^	0.17^**^	0.19^*^	0.22^**^

CES-D, Center for Epidemiologic Studies Depression Scale; FIQ-R, Fibromyalgia Impact Questionnaire Revised; PCS, Pain Catastrophizing Scale; STAI-T, Trait Anxiety Inventory. n.s., non-significant

* p < 0.05;

** *p < 0.01*.

### Discriminant validity: differences in cognitive emotion regulation between FMS patients with vs. without clinically significant depression

More than three-quarters of our participants (84.4%) presented clinically relevant depressive symptoms. Student's *t* and χ^2^ tests revealed that the two subgroups (FMS + depression vs. FMS) were fully comparable in their demographic characteristics (including duration of illness). As shown in Table 5, the FMS patients with clinically relevant depression scored significantly higher on the Self-blame, Rumination, Catastrophizing, and Other-Blame subscales than the FMS participants without depression. The differences in Positive refocusing and Positive reappraisal were also significant, but in the opposite direction. The significant differences oscillated from medium to large in magnitude according to Cohen's criteria. Some null differences were obtained. Specifically, those patients that were depressed did not differ from the non-depressed subgroup on the Acceptance, Refocus on planning, and Putting into perspective subscales. Overall, our data on the criterion-related validity of the CERQ subscales support the FMS-relevance of some of the measured cognitive emotion regulation strategies for discriminating among patients with/without affective comorbidity. Means and standard deviations of the CERQ scales are shown in Table 5. For the sake of comparability, Table 5 also shows the descriptive CERQ data obtained in a sample of 615 Spanish students (Domínguez-Sánchez et al., 2013) and 99 Dutch patients with clinically relevant depression and anxiety (Garnefski et al., 2002). With the exception of Catastrophizing, it seems that FMS patients do not use the *a priori* less adaptive cognitive emotion regulation strategies (including Acceptance) more frequently when compared with non-clinical Spanish subjects. In contrast, with the exception of Putting into perspective, patients report having used the more adaptive strategies less often. Patients with FMS in our study that had clinically relevant depressive symptoms had similar CERQ subscale scores compared with patients referred for treatment at an outpatient psychiatric clinic in the Netherlands who had significant depressive and anxiety symptoms. These comparisons should be interpreted with caution due to the absence of statistical analyses and matching in relevant variables such as gender or age.

**Table 5:** Discriminant Validity: Subgroup Comparisons (FMS vs. FMS + Depression) on the CERQ Subscales in Subsample 1 (*n* = 160).

***CERQ Subscales*^¥^ (4–20)**	**Domínguez-Sánchez et al. (2013) *n* = 615 Spanish students**	**Garnefski and Kraaij (2007) *n* = 99 with Anx/Dep**	**FMS total *n* = 160**	**FMS *n* = 24**	**FMS + depression *n* = 136**	**Student's *t* FMS vs. FMS+DEP**	**Cohen's d (90%CI)**
Self-Blame	10.59 (2.65)	10.97 (4.21)	9.86 (4.34)	7.17 (2.58)	10.34 (4.42)	4.89^**^	0.75 (0.57–1.01)
Acceptance	13.24 (3.14)	11.68 (3.74)	14.06 (4.22)	12.79 (3.90)	14.28 (4.25)	1.60	–
Rumination	13.34 (3.49)	12.64 (4.04)	13.16 (4.48)	9.42 (3.50)	13.82 (4.32)	4.72^**^	1.05 (0.87–1.40)
Positive refocusing	10.87 (4.00)	9.21 (3.65)	9.36 (5.08)	13.17 (4.72)	8.69 (4.86)	4.18^**^	0.93 (0.46–1.14)
Refocus on planning	15.58 (3.25)	12.62 (3.86)	12.96 (4.32)	13.29 (3.93)	12.90 (4.39)	0.41	–
Positive reappraisal	15.21 (3.89)	10.19 (4.09)	11.66 (4.49)	13.67 (4.55)	11.30 (4.40)	2.42^*^	0.54 (0.08–0.72)
Putting into perspective	13.72 (3.89)	10.54 (3.86)	13.63 (4.02)	13.96 (4.48)	13.57 (3.95)	0.43	–
Catastrophizing	7.96 (2.98)	9.11 (4.19)	9.35 (4.26)	6.25 (2.36)	9.90 (4.29)	6.01^**^	0.90 (0.71–1.13)
Other-Blame	7.80 (2.53)	7.76 (3.55)	8.16 (4.76)	5.54 (1.93)	8.62 (4.96)	5.30^**^	0.66 (0.45–0.85)

¥ Data expressed as means (standard deviation). n.s. = non-significant

* p < 0.05;

** *p < 0.01*.

Finally, given that the two subgroups were almost identical in their sociodemographic characteristics, it was unnecessary to control for these variables in the subsequent logistic regression analysis. The regression model explained 24.9% of the total variance [$\chi_{\left(9\right)}^{2}$ = 45.88, *p* < 0.001]. The Wald statistic was used to determine the significance of the contribution of the independent variables and the standardized β to ascertain the relative influence of each independent variable. As can be seen in Table 6, only two cognitive emotion regulation strategies were independent predictors of subgroup membership: Positive refocusing (standardized β = 0.13) and Catastrophizing (standardized β = 0.22). Therefore, subgroup membership was related to higher reported use of Catastrophizing and lower reported use of Positive refocusing. A new logistic regression model was computed including the two significant predictors only. This model yielded a slightly lower percentage of total explained variance (16.9%), but both predictors remained significant.

**Table 6:** Identification of Cognitive Emotion Regulation Strategies Discriminating Subgroup Membership (FMS with vs. without Depression): Initial Logistic Regression Model and Final Logistic Regression Model (between brackets).

**Predictors**	**Standardized β**	**SE β**	**Wald**	***p***
Self-Blame	0.05	0.11	0.18	0.67
Acceptance	0.11	0.07	2.41	0.12
Rumination	0.12	0.08	2.14	0.14
Positive refocusing	−0.13 (−0.15)	0.06 (0.05)	4.20 (10.26)	0.04 (0.01)
Refocus on planning	0.01	0.08	0.01	0.92
Positive reappraisal	−0.06	0.08	0.55	0.46
Putting into perspective	−0.04	0.08	0.23	0.63
Catastrophizing	0.22 (0.28)	0.10 (0.09)	4.68 (10.35)	0.03 (0.01)
Other-Blame	0.20	0.11	3.12	0.08

*Total explained variance (Cox & Snell R^2^): 24.9%*.

*Total explained variance (Cox & Snell R^2^) of the final model (with the two significant predictors only): 16.9%*.

*Significance model: $\chi_{\left(\text{9}\right)}^{\text{2}}=45.88$, p < 0.001*.

*Significance of the final model (with the two significant predictors only): $\chi_{\left(\text{2}\right)}^{\text{2}}=$ 29.61, p < 0.001*.

## Discussion

The CFAs computed on the CERQ supported the original nine-factor model in a Spanish sample of adult patients with FMS. This factor solution best fit the data, which is consistent with previous published psychometric studies carried out in other countries. For instance, a sample of French-speaking, young community volunteers completed the CERQ in the study by Jermann et al. (2006). The principal component analysis (PCA) suggested extracting nine factors that explained 56.7% of the variance and the CFA with the maximum likelihood (ML) method supported the nine-factor model (CFI = 0.94; RMSEA = 0.06). As with our study, the authors also tested a second-order factor model with adaptive and less adaptive strategies which provided good fit to the data. Similarly, Zhu et al. (2008) examined the dimensionality of the CERQ in Chinese university students performing a CFA with ML as the estimation method. The first-order nine-factor model fit the data well (CFI = 0.91, NNFI = 0.9, RMSEA = 0.05). More recently, Megreya et al. (2016) analyzed the psychometric properties of the Arabic version of the CERQ in four Arabic-speaking Middle Eastern countries (Egypt, Kingdom of Saudi Arabia, Kuwait, and Qatar). In line with our study, due to the ordinal nature of the items, the WLSMV estimator was used in the CFA. Overall, the goodness-of-fit indices indicated a good fit of the nine-factor model in the cases of Egypt, Kingdom of Saudi Arabia, and Qatar. The subsequent second-order CFA for each country, yielded poorer fit for the four countries in all indices compared with the first-order factor models. Therefore, the accumulated empirical evidence suggests that the first-order nine-factor structure is retained beyond the cultural context.

Inspection of the specific item-loadings is also in line with previous factor analytic studies performed on the CERQ. However, studies of different cultural versions of the CERQ have reported low or null standardized factor loadings for some items. For example, Domínguez-Sánchez et al. (2013) reported factor loadings of −0.11 for item 19 and −0.10 for item 20. Similarly, the PCA conducted by Jermann et al. (2006) indicated that the maximum loading of each CERQ item was found on the assigned factor, except for items 19 and 20. The saturation of item 8 on its factor was below 0.3. In contrast, we found that all 36 items could be retained taking common cut-off criteria for item retention into account. The lowest factor loading was 0.55 in the present work (item 8 in the original form). In our opinion, the main reason of this increase in factor loadings in our study is that items were grouped by factor. Our change in item presentation, taking the possible impact of *fibrofog* (Katz et al., 2004) into account, may have facilitated patients' responses to items as a result of a clearer understanding of what the four items per dimension are attempting to measure (Schell and Oswald, 2013). Further studies are needed to discern in which evaluation circumstances and for whom item grouping or item randomization is most recommended.

All CERQ subscales showed high internal consistency, ranging from 0.77 (Acceptance) to 0.93 (Positive refocusing) and, with minimal exceptions, were null or modestly correlated with each other, indicating that some subscales share common variance but also represent unique dimensions. Only Rumination and Self-blame presented a large correlation (>0.5). In general, the Cronbach's alphas and subscale correlations found here do not differ from those reported by other authors (e.g., Garnefski et al., 2002; Jermann et al., 2006; Zhu et al., 2008; Tuna and Bozo, 2012; Ireland et al., 2017). When 396 Turkish university students completed the Turkish version of the CERQ, Tuna and Bozo (2012) observed that the subscales were relatively independent with a mean correlation coefficient of 0.2. Internal consistency of the subscales ranged between 0.72 (Self-blame) and 0.83 (Catastrophizing). In a clinical adult population with symptoms of depression and anxiety (Garnefski et al., 2002), Cronbach's alpha values for the CERQ ranged from 0.72 (Acceptance) to 0.85 (Self-blame). We consider it particularly important in our case to establish comparisons because psychometric evidence of the CERQ has mainly been obtained in non-clinical samples composed of healthy community adults or university students.

Although we could not establish causal relationships due to the cross-sectional nature of our data, it is reasonable to infer that some specific cognitive emotion-regulation strategies might be considered risk factors for or protective factors against depressive and anxiety symptoms and functional status in patients with FMS. The following findings are noteworthy. The strategies Refocus on planning and Putting into perspective had non-significant correlations with functional status, pain catastrophizing, depressive symptoms and trait anxiety. The strategies of Catastrophizing, Rumination, and Self-blame emerged as counterproductive strategies. Positive refocusing negatively correlated with the aforementioned pain-related and psychological variables and, finally, Acceptance and Positive reappraisal had relatively small relationships with these variables. In fact, the apparently counterintuitive positive significant correlation between Acceptance and the pain-related and psychological variables is not surprising. Jermann et al. (2006) pointed out that items related to thoughts of acceptance and resignation are mixed up within this strategy. From a clinical perspective, Acceptance is considered to be an adaptive strategy whereas resignation is similar to helplessness. Higher Acceptance measured with the CERQ has been found to be positively associated with higher depressive symptoms in both Chinese and North-American samples (Martin and Dahlen, 2005; Zhu et al., 2008). Acceptance exhibited significant positive correlations with general symptoms of psychopathology in a Turkish sample (Tuna and Bozo, 2012). Even the designers of the instrument found that Acceptance had significant positive relationships with depressive symptoms in a general adult sample and in the elderly (Garnefski and Kraaij, 2006). Thus, taking the body of literature and our higher-order factor models into account, we can conclude that Acceptance (as measured in the full version of the CERQ) cannot be considered as part of the repertoire of adaptive cognitive emotion regulation strategies. We agree with Martin and Dahlen (2005, p. 1256) when they stated that “*the presumably adaptive role of acceptance needs to be reconceptualised*.”

In addition, we were interested in analyzing whether frequency of use of *a priori* adaptive and less adaptive emotion regulation strategies was influenced by the presence of comorbid depression. FMS patients with clinically relevant depression were expected to use less adaptive strategies more frequently than those patients without comorbid depressive symptoms. We used a CES-D cut-off to dichotomize the FMS sample (depressed vs. non-depressed). Although it is well-known that splitting a variable into categories results in loss of information and might increase the probability of type II errors (Altman and Royston, 2006), we observed additive effects of depression, that is, the relationship between FMS and cognitive emotion regulation was influenced by the presence of clinically relevant depressive symptoms. Specifically, those participants suffering clinically relevant depression reported more frequent use of Self-blame, Other-blame, Rumination, and Catastrophizing and less use of Positive refocusing and Positive reappraisal, which is clinically coherent. The subsequent regression analyses revealed that Catastrophizing and Positive refocusing were the strategies that significantly discriminated between patients with/without depression. Bearing in mind the high prevalence of clinically relevant depressive symptoms detected in our sample and that depression is characterized by impaired emotion regulation (Joormann and Stanton, 2016), the innovative *Emotion Regulation Therapy* (ERT; Mennin and Fresco, 2014; Renna et al., 2017) might be a potential add-on treatment for patients with FMS plus co-occurring depression. Originally developed for generalized anxiety disorder comorbid with major depression, ERT is a transdiagnostic mechanism-targeted treatment for distress disorders, which makes it an interesting therapeutic option for FMS, a distress-related disorder according to some specialists in this syndrome (Schweinhardt et al., 2012).

Our study is limited by the use of self-report measures and by its cross-sectional nature, which prevents causal inferences and the assessment of important psychometric aspects such as test-retest reliability, sensitivity to change, or longitudinal prediction of clinically relevant and pain-related constructs. Moreover, assessment of the habitual use of cognitive emotion regulation strategies relies on recall, which may be particularly problematic for strategies whose use is highly contextually dependent, such as Acceptance or Positive reappraisal. In addition, due to the predominance of women among participants, we were not able to examine gender differences in the use of CERQ strategies, as has been done in many previous studies carried out in Western, Middle Eastern, and Eastern countries (Martin and Dahlen, 2005; Megreya et al., 2016). We did not implement statistical techniques to mitigate potential “method biases” (Podsakoff et al., 2012) in our data because we judged that our participants were able to provide accurate answers. In fact, the CERQ items were grouped by dimension in the present work, a change in item presentation that facilitates responses as a result of a clear understanding of what the items related to each dimension are attempting to measure. Moreover, method biases are less likely in respondents that are motivated to provide optimal responses to the items. Patients with FMS have a strong desire for self-expression, CERQ items imply intellectual challenge and in part some emotional catharsis; and patients have the desire to help clinicians improve available treatments for their condition. In summary, stylistically or non-differentiated responding was not expected a priori.

To sum up, our findings indicate that the CERQ is a sound instrument for assessing cognitive emotion regulation in patients with FMS and the reported results add to several previous studies that have found a consistent association between cognitive emotion regulation strategies and depressive-anxious symptoms across countries and across clinical and non-clinical samples.

## Author contributions

AF-S and JL made substantial contribution to the analysis and to the interpretation of the data, drafted the manuscript, provided final approval of the version to be published, and agreed to be accountable for all aspects of the work in ensuring that questions related to the accuracy or integrity of any part of the work are appropriately investigated and resolved. ER-C, XB, and MP-M made substantial contributions to the conception and the design of the study, drafted the manuscript, provided final approval of the version to be published, and agreed to be accountable for all aspects of the work in ensuring that questions related to the accuracy or integrity of any part of the work are appropriately investigated and resolved. AP-A and LA-R helped out in the interpretation of data for the work, revised the manuscript critically for important intellectual content, provided final approval of the version to be published, and agreed to be accountable for all aspects of the work in ensuring that questions related to the accuracy or integrity of any part of the work are appropriately investigated and resolved. MN-G, JG-C, and JB helped out in the interpretation of data for the work, revised the manuscript critically for important intellectual content, provided final approval of the version to be published, and agreed to be accountable for all aspects of the work in ensuring that questions related to the accuracy or integrity of any part of the work are appropriately investigated and resolved.

### Conflict of interest statement

The authors declare that the research was conducted in the absence of any commercial or financial relationships that could be construed as a potential conflict of interest.

## Footnotes

^1^As suggested by one anonymous reviewer, a bifactor structure was also fitted, examining whether the CERQ could be modeled using two general factors of ‘adaptive’ and ‘less adaptive’ strategies, as measured by a priori adaptive and less adaptive items, respectively and nine specific factors, as measured by item subsets. A bifactor approach (Rodriguez et al., 2016) helps to determine whether the CERQ items are multidimensional, allowing the computation of sub-scale scores, or whether the items are mainly unidimensional, for which only two total scores should be computed and reported (one total ‘adaptive’ score + one total ‘less adaptive’ score). Unfortunately, this model had estimation problems (empirically unidentified) that preclude its reporting in the manuscript as potential factor solution for the CERQ.
